# The Diagnosis and Management of Recurrent Gallstone Ileus: A Case Report

**DOI:** 10.7759/cureus.27978

**Published:** 2022-08-13

**Authors:** Mohammad Mishan, Bahareh Mehdikhani

**Affiliations:** 1 General Surgery, Abadan University of Medical Sciences, Abadan, IRN; 2 Radiology, School of Medicine, Iran University of Medical Sciences, Tehran, IRN

**Keywords:** cholecystoduodenal fistula, enterolithotomy, gallstone disease, bowel obstruction, gallstone ileus

## Abstract

Gallstone ileus is one of the rare and insidious causes of small bowel obstruction, which should always be kept in mind by clinicians, especially when encountering older people with a history of gallstones disease. The high mortality and morbidity rate associated with the condition can be mostly attributed to delayed or misdiagnosis. Imaging modalities, particularly CT scans, play an important role in correct and timely diagnosis. We present the case of a 65-year-old man with a two-year history of colicky biliary pain, who had severe abdominal pain with obstructive symptoms for five days before admission. The diagnosis of gallstone ileus is made using CT scan findings. Enterolithotomy alone was performed three days later. A second, smaller migrated gallstone was also found, which excreted spontaneously. After 20 days, the patient achieved full recovery and was discharged.

## Introduction

Gallstone ileus is defined as small bowel mechanical obstruction induced by an impacted ectopic gallstone. Terminal ileum and ileocecal valve are the most common sites of gallstone impaction. Generally, gallstones of less than 2-2.5 cm have a high probability of spontaneous transit [1]. Gallstone ileus is a rare complication of long-term cholelithiasis. The risk factors include female gender, age over 65 years, history of recurrent episodes of untreated cholecystitis, and larger stones (>2.5 cm) [2].

Inflammatory changes, pressure erosion, and ischemia of the gallbladder wall could lead to biliary-enteric fistula in patients with a long-standing history of gallstone disease. The most common fistula is cholecystoduodenal fistula (32.5-96.5%) followed by stomach, small bowel, and colon involvement, respectively [3].

Strong clinical suspicion is necessary for the early diagnosis of this condition. Nonspecific and intermittent symptoms, misdiagnosis, or delayed diagnosis are factors that cause high morbidity (20-57%) and mortality (7-18%) rates in this condition compared to other causes of small bowel obstruction [4]. The median duration of symptoms onset and diagnostic delay is six days. Delayed diagnosis leads to complications including dehydration, shock, sepsis, or peritonitis [5].

Radiological imaging is the cornerstone of the early diagnosis of gallstone ileus. The Rigler’s triad including pneumobilia, small bowel obstruction, and ectopic gallstone represent the pathognomic findings [6]. The treatment options include surgical (one-stage or two-stage closure of fistula) or conservative (natural closure of fistula) depending on the patient's clinical conditions [7].

## Case presentation

A 65-year-old male presented to the emergency department with a five-day history of constipation, nausea, mild abdominal pain, and two-year history of multiple episodes of colicky biliary pain controlled by painkillers and opium. He had experienced acute severe right upper quadrant pain seven days before admission. The pain had not responded immediately to painkillers; it had subsequently improved but he had developed intestinal subocclusion symptoms. He had a history of diabetes, hypertension, and opium addiction.

The patient's vital signs were stable; a non-tender abdomen and mild generalized distention were evident on physical examination. Lab data revealed leukocytosis (WBC=13000) and elevated creatinine level (Cr=2.1).

**Figure 1:**
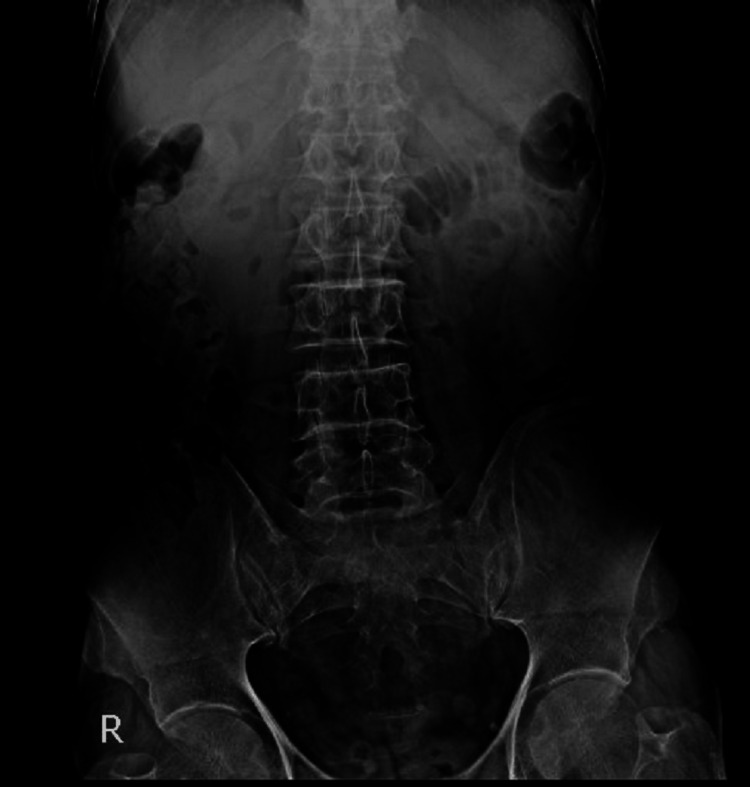
Upright abdominal X-ray showing mildly dilated small bowel loops

**Figure 2:**
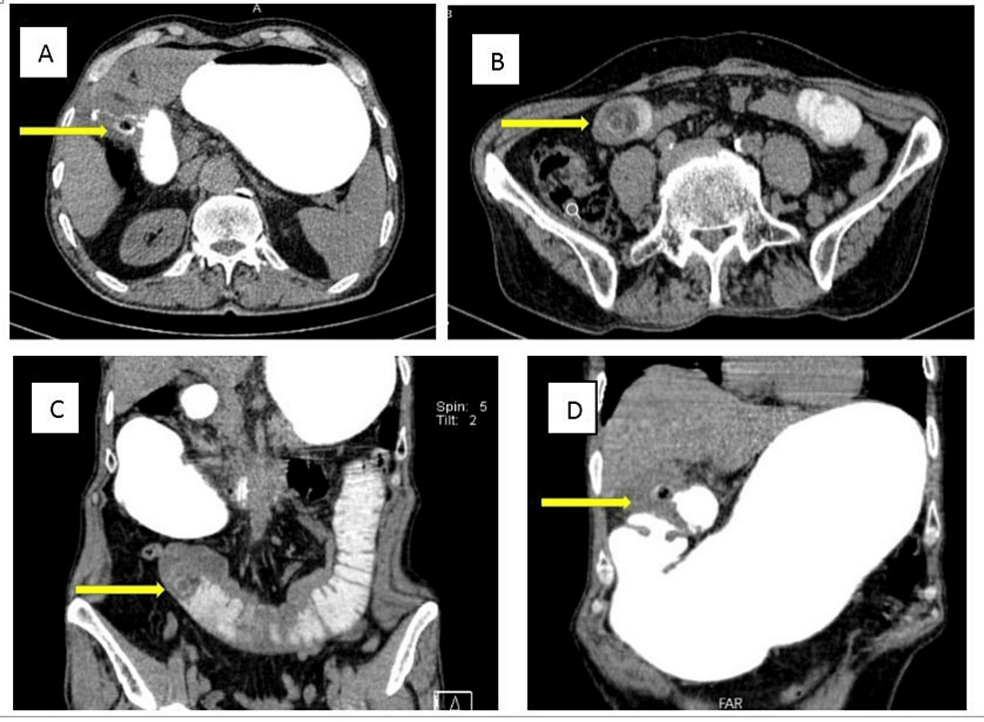
Oral contrast-enhanced CT - 1 The images demonstrated dilated jejunal loops (B, C), fistulous tract between gallbladder wall and duodenal bulb (A, D), oral contrast leakage to gallbladder lumen (A), obstructing layering gall bladder stone in the jejunum (B, C) compatible with gallstone ileus CT: computed tomography

Upright abdominal X-ray showed mildly dilated small bowel loops (Figure 1). An abdominopelvic CT scan with oral contrast was performed. Findings were compatible with gallstone ileus: dilated duodenal and jejunal loops, fistulous tract between gallbladder wall and duodenal bulb, oral contrast leakage to gallbladder lumen, obstructing layering gall bladder stone in the jejunum, and at least two other stones in the gall bladder (Figure 2).

Due to poor patient compliance and surgery refusal, as well as partial obstructive symptoms, conservative management was chosen at first, inevitably. But his symptoms and signs worsened during the first three days of admission, including bilious vomiting, obstipation, increasing abdominal pain, distention, and leukocytosis in favor of complete obstruction. Finally, the decision was made to perform an urgent laparotomy after getting informed consent.

The intraoperative examination showed dilated duodenal and jeujonal non-ischemic loops, impacted jejunal stone at the transitional point, and adhesions over the gallbladder and duodenal wall. An enterotomy was done using a longitudinal incision. An oval-shaped stone measuring 28 x 25 mm and biliary material were extracted, and then the double layer closure of enterotomy was performed. The remainder of the gastrointestinal tract was assessed carefully to find other ectopic stones, but none were found. The area of the cholecystoduodenal fistula was not manipulated due to the patient's condition and potential risk of injury due to extensive adhesions.

**Figure 3:**
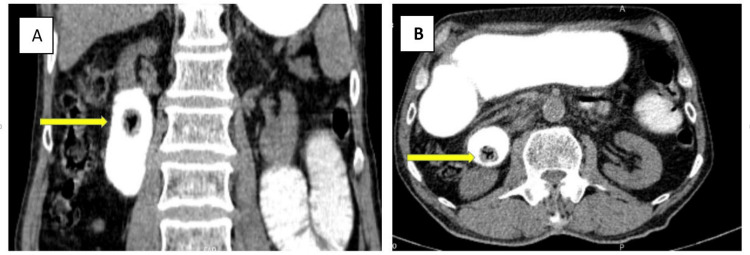
Oral contrast-enhanced CT - 2 The images revealed a recent duodenal ectopic non-obstructing gallbladder stone with a typical Mercedes-Benz sign (A, B) CT: computed tomography

On the fifth postoperative day, after beginning an oral diet, mild colicky abdominal pain and bilious vomiting were revealed. A second abdominopelvic CT scan with oral contrast was performed, which demonstrated another duodenal ectopic non-obstructing gallbladder stone measuring 17 x 15 mm with a typical Mercedes-Benz sign in favor of recently migrated gall stone (Figure 3). Since the patient had no obstructive symptoms, conservative treatment and follow-up were opted for.

During the next seven days, the patient did not experience any obstructive symptoms and signs and tolerated the semi-liquid diet well, which was suggestive of spontaneous excretion of the stone, subsequently confirmed by abdominopelvic CT. The patient experienced a full recovery and was discharged 20 days after the admission.

## Discussion

Gallstone ileus is defined as small bowel mechanical obstruction induced by an impacted ectopic gallstone [1]. Nowadays, gallstone ileus is a very rare entity due to the early diagnosis and treatment of the gallstone disease [8]. The incidence of gallstone ileus is 0.3-0.5% in patients with cholelithiasis and it is responsible for 1-5% of all cases of mechanical bowel obstructions and 25% in individuals older than 65 years. The most affected age group is over 65 years with a higher prevalence among females (3:1) [9,10].

The terminal ileum and the ileocecal valve are the most common anatomical sites for gallstone impaction because of their small caliber and less active movements. Ileum (60.5% of cases), jejunum (16.1%), stomach (14.2%), colon (4.1%), and duodenum (3.5%) can also get involved. It can also pass spontaneously in 1.3% of cases [10-12]. In our case, the site of impaction was the jejunal loop.

Inflammatory changes, pressure erosion, and ischemia of the gallbladder wall could lead to biliary-enteric fistula in patients with a long-standing history of gallstone disease. Cholecystoduodenal fistula is the most common manifestation (32.5-.96.5%) [3] as shown in our case, which was well demonstrated in the CT scan.

The classic presentation of gallstone ileus entails a “tumbling obstruction” in an elderly woman, indicating periodic subacute obstruction. Episodic and transient gallstone impaction results in nonspecific, generalized abdominal pain and vomiting. In our patient, a smaller migrated gallstone was also found, which, under our careful observation, resembled a “tumbling obstruction” and spontaneously resolved. The minimum size of the ectopic stone that leads to the development of obstruction is 2.5 cm in diameter. In the study by Clavien et al., the size of obstructing gallstone ranged from 2 to 5 cm [9]. Multiple gallstones have been reported in 3-40% of cases, which were found to be responsible for recurrent gallstone ileus episodes [11].

Nonspecific and intermittent signs and symptoms, the advanced age of the patients and their comorbidities, and misdiagnosis or delayed diagnosis are factors that cause high morbidity (20-57%) and mortality (7-18%) rates in this condition compared to other causes of small bowel obstruction [4]. The median duration of symptoms onset and diagnostic delay is six days. Delayed diagnosis can lead to complications including dehydration, shock, sepsis, or peritonitis [5].

Radiological imaging is the cornerstone for the early diagnosis of gallstone ileus. The classic Rigler’s triad illustrates the radiographic features of gallstone ileus: small bowel obstruction, pneumobilia, and an ectopic gallstone that migrates in serial films through the GI tract; the presence of two of them is enough for gallstone ileus diagnosis. Only 10% of gallstones are radiopaque, and this along with the presence of fecal material renders plain abdominal radiography incapable of detecting gallstones and reduces the chance of an early diagnosis [13]. Ultrasound helps to make a correct diagnosis by detecting cholelithiasis, fistulous tract, and pneumobilia. Interestingly, no aerobilia detected by ultrasound may have a role in delayed diagnosis [14,15]. Enhanced CT scan is the most effective diagnostic modality in terms of sensitivity and specificity (93% and 100% respectively), and it is associated with several benefits, such as determining the viability of the involved segment of the bowel preoperatively and providing information for a decision-making strategy and therapeutic approach [16].

Lassandro et al. found that the Rigler’s triad is observed in 14.8% of cases via plain abdominal radiography, 11.11% of cases on ultrasound examination, and 77.78% on abdominal CT scan [14]. In our patient, the Rigler's triad was present on the CT scan, but it was not clearly visible on the abdominal X-ray. A correct preoperative diagnosis has been reported only in 50% of cases. Sometimes, gallstone ileus is diagnosed during surgery in patients with an unknown cause of small bowel obstruction [17].

The definitive treatment involves the removal of the impacted stone and relieving intestinal obstruction subsequently. However, preoperative conditions such as the age of the patients, comorbidity, diagnostic delay, and the need for urgent surgery have a significant impact on choosing the best approach, which should be decided by a careful evaluation of the risk-to-benefit ratio [18].

Three surgical methods are usually employed for gallstone ileus treatment: enterolithotomy alone, enterolithotomy with cholecystectomy and simultaneous fistula closure (one-stage procedure), enterolithotomy and delayed cholecystectomy, typically four to six weeks later (two-stage procedure) [7].

Enterolithotomy alone is the most commonly used method. An enterotomy involves extracting the stone through a longitudinal incision. The rest of the bowel is then evaluated for other migrated stones. Eventually, the incision site is sutured in a transverse fashion [3,15,16].

The one-stage procedure is suitable for low-risk patients without comorbidity, those who are relatively younger, and those with less severe disease, adequate nutrition, and stable hemodynamics, owing to the longer duration of surgery, postoperative complications, and hospitalization [3,15]. The one-stage procedure is associated with higher mortality rates than simple enterolithotomy (16.9 vs. 11.7%). It was found that biliary symptoms remained in 15% of patients managed with simple enterolithotomy; however, only 10% required further surgery to relieve symptoms. The recurrence rate of gallstone ileus in this group was about 5%, usually occurring within six months of initial presentation. Despite these risks, spontaneous fistula closure occurs in 50% of cases [19].

The two-stage method carries the risk of cholecystitis, recurrence, and gallbladder malignancy due to the continuation of the bilioenteric fistula This procedure is recommended in high-risk patients who suffer from persistent symptoms secondary to residual gallstones or biliary fistula. The risk of gallstone ileus recurrence with a two-stage approach is 5-9% and only 10% of them require another surgery [15]. Chou et al. reported migration of multiple small gallstones after surgery in their study, but only 10% of these patients required reoperation. Hence, facing the real risk of re-do with a higher operative mortality rate compared to enterolithotomy should be considered when opting for this procedure [13].

Conservative management is also useful in some cases. Indeed, when gallstones are only up to 2.5 cm in size, they can be discharged spontaneously. However, conservative treatment cannot always be recommended, because it leads to a positive result only in cases of partial intestinal obstruction [11,20].

As mentioned before, our patient underwent enterolithotomy alone, and the main reasons for preferring this approach were severe inflammatory changes and many adhesions around the gallbladder, which increased the risk of iatrogenic injuries. Also, the patient's advanced age, presence of comorbidities, and poor compliance played a role in this decision. In the case of the second ectopic stone, which was detected in the follow-up CT scan, we preferred conservative treatment over re-enterolithotomy due to the stone size and absence of clear obstruction signs, and it passed through the GI tract, fortunately. CT scan had a significant role in our decision-making, as it enabled us to estimate the size and location of the stone and choose the non-surgical option for treatment.

## Conclusions

Nowadays, the advanced manifestations of gallstone disease have considerably declined due to early diagnosis and treatment. Gallstone ileus should be considered when encountering a case of small bowel obstruction, especially in elderly individuals with a positive history of cholelithiasis. The best surgical option for gallstone ileus is still a subject of controversy. Due to the low risk of complications, enterolithotomy alone is the most popular surgical procedure. In our case, the delay in surgery due to patient refusal, his general clinical condition, the patient's advanced age, as well as extensive adhesion and inflammatory changes of the gallbladder and the duodenal wall prompted us to opt for enterolithotomy alone. CT scan is a readily available, quick, and high-resolution diagnostic modality, which, due to its high diagnostic accuracy, plays a decisive role both in the diagnosis and in determining the treatment plans. In our case, the findings of the CT scan enabled us to avoid performing re-surgery.
